# HBV and HCV testing outcomes among marginalized communities in Italy, 2019–2024: a prospective study

**DOI:** 10.1016/j.lanepe.2024.101172

**Published:** 2024-12-09

**Authors:** Monica Monti, Teresita Caruso, Alice Castellaccio, Irene De Giorgi, Gabriella Cavallini, Maria Laura Manca, Serena Lorini, Silvia Marri, Luisa Petraccia, Francesco Madia, Cristina Stasi, Laura Carraresi, Elisabetta Lorefice, Sara Irene Bonelli, Alessandro Nerli, Mouheb M.A. Mudalal, Lorenzo Martini, Stefano Gitto, Eleonora Carradori, Adela Xheka, Irene Bendini, Samuele Lukolic’, Lorenzo Latella, Donatella Aquilini, Pierluigi Blanc, Anna Linda Zignego, Laura Gragnani

**Affiliations:** a MASVE Interdepartmental Hepatology Center, Department of Experimental and Clinical Medicine, University of Florence, Center for Research and Innovation CRIA-MASVE, Firenze, Italy; b Department of Translational Research and of New Surgical and Medical Technologies, University of Pisa, Pisa, Italy; c San Jacopo Hospital, Infectious Disease Unit, Pistoia, Italy; d Department of Clinical and Experimental Medicine and Department of Mathematics, University of Pisa and University Hospital of Pisa, Pisa, Italy; e Department of Neuroscience, Psychology, Drug Research and Child Health (NEUROFARBA), Section of Pharmacology, University of Florence, Florence, Italy; f Poison Control Center Unit, Department of Emergency, Anesthesia and Critical Care Medicine, Policlinico Umberto I Hospital - Sapienza, University of Rome, Rome, Italy; g Regional Health Agency of Tuscany, Florence, Italy; h Department of Life Science, Health, and Health Professions - Link Campus University, Roma, Italy; i San Giuseppe Hospital, Gastroenterology Unit, Empoli, Italy; j Santo Stefano Hospital, Infectious Disease Unit, Prato, Italy; k Department of Health Sciences, University of Florence, Florence, Italy

**Keywords:** Hepatitis B virus, Hepatitis C virus, Marginalized populations, Healthcare disparities, World Health Organization

## Abstract

**Background:**

The health of the marginalized populations is crucial for public health and inequalities. The World Health Organization (WHO) Global Hepatitis Report 2024 stated that over 304 million people were living with Hepatitis B Virus (HBV)/Hepatitis C Virus (HCV) infection in 2022. We performed HBV/HCV screenings among marginalized communities to reveal hidden infections and link-to-care positive participants.

**Methods:**

From January 2019 to May 2024, finger-prick tests were used to conduct on-site screenings at non-profit organizations in Tuscany, Italy. Positive participants were referred to the closest outpatient clinic.

**Findings:**

Eighty/1812 (4.4%) participants were Hepatitis B surface Antigen (HBsAg)+, mostly men (*p* < *0.001*) and non-Italian natives compared to those HBsAg- (*p* < *0.001*). Fifty-two/1812 (2.9%) were anti-HCV+ with a higher proportion of Italians (*p* < *0.001*) and lower education level (*p* < 0.01) compared to the anti-HCV-. Intravenous drug use was an independent factor for being anti-HCV+ (*p* < *0.0001*). Among the HBsAg + individuals, 66.3% (53/80) were linked and 90.4% (48/53) retained in care (treated/monitored). Of the anti-HCV participants requiring clinical evaluation, 37.8% (14/37) were linked to care, and all the 11/14 (88.6%) viremic patients were successfully treated.

**Interpretation:**

We found higher HBV/HCV positivity compared to national prevalences. Participation and linkage to care were successful. The young mean age (33.6 yrs) of HBsAg + individuals, primarily from regions with low vaccinal adherence, indicated geographical origin as a key risk factor. HCV positivity was associated with extreme marginality. The results stress the need to implement marginalized groups screening to target HBV/HCV hidden infections, reducing disparities in healthcare and advancing towards the WHO 2030 elimination goal.

**Funding:**

Gilead Sciences; 10.13039/100007387Fondazione Cassa di Risparmio di Pistoia e Pescia; 10.13039/501100009888Regione Toscana.

**Research in context**

**Evidence before this study**

As of May 2024, a PubMed search was conducted using the queries “Hepatitis B Virus (HBV) screening or testing”, “Hepatitis C Virus (HCV) screening or testing” “marginalized/underprivileged/vulnerable populations” and “viral hepatitis elimination”. The search revealed several studies focused on marginalized populations, predominantly conducted in the USA. Those performed in Europe often included small sample sizes or were conducted before the COVID-19 pandemic. Studies carried out in Italy primarily employed testing strategies targeting specific settings (mainly migrants) and usually did not address marginalized Italian natives.

Overall, as previous reports have shown, the typical HBV and HCV screening strategies adopted in Europe to meet the World Health Organization’s (WHO) goal of eliminating HBV and HCV as a public health threat by 2030 often do not include disadvantaged populations due to challenges in outreach, linkage to care, and low compliance. This exclusion hampers the viral hepatitis elimination plan and exemplifies health inequality, particularly among marginalized groups such as migrants and homeless people, a topic of growing concern for the public health community.

**Added value of this study**

The higher HBV/HCV prevalence we found in marginalized settings compared to the general Italian population highlights the need to expand screening efforts to vulnerable groups to target residual/hidden infections. This would reduce disparities in access to treatment and help progress toward the WHO’s goal to end viral hepatitis as a public health threat by 2030. Our findings suggest that an integrated on-site testing strategy, involving navigators and social workers, is likely the key to achieving high adherence, linkage, and retention in the care of positive cases, even without financial incentives for participation. The results also indicate a difference between HBV and HCV positive subgroups: while HBV is more prevalent among economic migrants and refugees temporarily living in shelters, who do not typically experience social exclusion in their home countries, the profile of the HCV-positive individual is one marked by extreme marginalization, risky behaviors, and a lack of health-related priorities. These insights could help in better targeting testing interventions.

**Implications of all the available evidence**

Our study demonstrates that the integrated on-site testing model we adopted could serve as a useful framework for policymakers tasked with reassessing existing health policies and systems related to viral hepatitis elimination at national and European levels. Additionally, Italy is a first port of call for many migrants seeking to reach the European Union. The provision of prompt HBV/HCV screening interventions could facilitate clinical management in their final destination countries enhancing individual health and social status. To achieve global HBV and HCV elimination, it is essential to develop strategies that improve healthcare accessibility while building trust and engagement with marginalized communities.

## Introduction

Hepatitis B Virus (HBV) and Hepatitis C Virus (HCV) are the most common causes of chronic liver disease worldwide.^1^ The Global Hepatitis Report 2024, recently released by the World Health Organization (WHO), states that over 304 million of people were living with HCV or HBV infection in 2022,^2^ making HBV and HCV two of the top ten “infectious killers” globally.^1^ Almost 1.3 million people die every year, due to chronic diseases caused by HBV and HCV.^2^ Furthermore, viral hepatitis is responsible for liver cancer (hepatocellular carcinoma- HCC), that is the most common primary liver cancer and the third leading cause of cancer-related mortality worldwide.^3^

Because chronic liver disease is asymptomatic until the late stages, estimates suggest that 40–80% of affected people are not aware that they are infected.^4^ Due to the high prevalence of chronic liver disease in developing countries, they bear a higher burden of morbidity and mortality for hepatic causes.^4^ For instance, it is estimated that 5–10% of adults in East Asia and Sub Saharan Africa may be infected with chronic HBV infection.^4^ In 2016, the WHO announced the goal to eliminate viral hepatitis as a public health threat by 2030.^5^ As stated by the last report, this important accomplishment requires a global action although, currently, there are still several gaps in HBV and HCV elimination policies adopted by the countries.^2^

An effective strategy for HCV elimination is leveraging the concept of “micro-elimination”.^6^, ^7^, ^8^ Micro-elimination is a targeted approach aimed at reducing HCV infection rates within specific high-risk groups, geographic areas, or settings rather than attempting to eliminate the virus across entire populations at once. These focused interventions are designed to address each group's unique challenges and needs, ultimately contributing to the broader global HCV elimination goals set by WHO.^6^^,^^9^^,^^10^ Micro-elimination approaches should adhere to specific criteria, although these may need adjustment based on epidemiological contexts and geographic locations.^6^ While this strategy was successfully used for HCV infection, there are few examples of micro-elimination studies regarding HBV.^11^^,^^12^

In addition, disparities in care and outcomes among underserved populations such as the homeless, marginalized people, and migrants are of growing interest to public health. The prevention, identification, and timely management of infections caused by HBV and HCV, are necessary health interventions to provide health care that is accessible to all. These interventions are essential in improving the living conditions of the vulnerable people. It will aid in ceasing the spread of diseases due to HBV and HCV that are intended to be eliminated in the future, in accordance with the WHO directives.^2^^,^^5^

Here, we report results from a screening project aimed at the identification and linkage to care of Hepatitis B surface Antigen (HBsAg) and anti-HCV positive individuals. The project was conducted in the metropolitan areas of Florence, Prato, and Pistoia, three cities in Tuscany, a central Italian region. Testing campaigns were performed on-site in different local charities, such as meal centers, shelters for migrants, and help centers for marginalized people.

## Methods

A prospective study to identify and, when necessary, treat HBV and HCV infection was carried out by performing on-site screening sessions at different charities and shelters operating in the metropolitan areas of Firenze, Prato and Pistoia, Tuscany, Italy. The sites included non-profit organizations providing support to those in need (charities), such as meal centers, shelters for migrants, food banks, Italian language schools for foreigners, and religious gathering places. The charities operating in the area covered by the study were first contacted by phone or email to schedule an in-person or online meeting to explain the objectives and methods of the screening project in detail. Community navigators at the non-profit organizations that agreed to host the testing sessions, received specialized training to provide charity users with accurate information about the testing. Posters and leaflets announcing the upcoming sessions were available throughout the charities at least two weeks before the screenings.

All the charity users ≥18 years of age were eligible for testing unless the presence of psychiatric disorders or language barriers that would prevent from correctly understanding of the testing aims, consenting or participation in the study. Cultural mediators were recruited for the sessions at shelters for immigrants (mostly Urdu, Arabic and Bengali speakers, depending on the geographical origin of the individuals attending the charity). Healthcare personnel involved in the screening could speak French, English, Albanian and Levantine Arabic. The number of cultural mediators and navigators depended on the number of users/charity.

The healthcare personnel team included a physician, three researchers and a nurse. After a short interview to collect a brief medical history and inform the participants about the screening’s aim, those who agreed to be tested signed an informed consent form. The form was available in different languages (Italian, English, French, Chinese, Albanian, Arabic and Spanish).

The interview was useful in determining possible risk factors for HBV and HCV infection such as previous or current use of intravenous (IV) injected drugs, unprotected sex, familial exposure, previous surgeries or blood transfusions. Questions about comorbidities and education level were also included in the survey.

Rapid Diagnostic tests (RDT) for HBsAg (Abbott Rapid Diagnostics, USA; sensitivity: 97.2% [95% CI: 93.1, 99.2]; specificity: 98.5% [95% CI: 95.7, 99.7])^13^ and anti-HCV positivity (Hangzhou All Test Biotech Co., Ltd, China, distributed by ScreenItalia, Italy; sensitivity: 100% ([95% CI:99:4%–100%]; specificity: 100% [95% CI:99: 8%–100%]) were performed on whole blood obtained via a finger prick.

HBsAg and anti-HCV positive participants were advised to attend a further evaluation appointment at the nearest outpatient clinic that had previously agreed to participate in the study. The healthcare personnel were able to make appointments immediately after communicating the test results or, at the latest, the day after, to provide easy and quick access to care. Positive participants were referred to the following outpatient clinics of the centers participating in the study: MaSVE Interdepartmental Hepatology Center, University of Florence, Florence; Infectious Disease Unit, “San Jacopo” Hospital, Pistoia; Infectious Disease Unit, “Santo Stefano” Hospital, Prato; Gastroenterology Unit, “San Giuseppe” Hospital, Empoli.

Linkage to care consisted of clinic appointments, assessment of viremia, liver function tests and where possible, a liver FibroScan (Echosens, Paris, France). An abdomen ultrasound was also performed at the initial appointment. According to a previous study,^14^ two different linkages to care outcomes were assessed: linked to care (attended one clinic appointment) and retained in care (attended >1 clinic appointment), meaning that patients were monitored or received therapy, based on the evaluation of clinicians. HBV and HCV treatments offered were in accordance with the European guidelines.^15^^,^^16^

HBsAg negative participants were referred to the local disease control and prevention center to assess whether they were susceptible to HBV vaccination.

The Ethical Committee of Area Vasta Centro provided ethical approval for this study (evaluation code: 17886_bio, updated after the COVID-19 pandemic -10/18/2022- with the new code 22562_spe). All research was conducted in accordance with both the Declarations of Helsinki and Istanbul. All participants gave written consent.

### Statistical analysis

The primary endpoints, i.e., the prevalence of HBsAg and anti-HCV, were detected by rapid tests. Missing data (regarding unprotected sex and education level) were excluded from the analysis. Categorical variables were presented using frequencies and percentages. Continuous variables were summarized as mean (SD) if normally distributed (assessed by Shapiro–Wilk test) or median (IQR) otherwise.

Differences in continuous variables were assessed using Student t-tests or Mann–Whitney U tests, as appropriate, while categorical variables were compared using χ^2^ or Fisher's exact tests. In the case of more than 2 categories and *p* was significant on the χ^2^ test, the residuals were analyzed to see which categories contributed to significance.

Two multivariate logistic regression models were fitted, one for HBV positivity and one for HCV positivity. Independent variables included sex, age, comorbidities (e.g., diabetes, hypertension), and risk factors (e.g., intravenous drug use, blood transfusion). Odds ratios (ORs) and 95% confidence intervals (CIs) were calculated to assess the strength of the associations.

If necessary, the *p* level was set at 0.05, adjusted for multiple comparison. Statistical analyzes were performed using Stata/SE version 17.0 (StataCorp, College Station, TX, USA) and R version 4.4.1.

World regions were classified according to the United Nation Geoscheme.^17^

### Role of the funding sources

Gilead Sciences; Fondazione Cassa di Risparmio di Pistoia e Pescia; Regione Toscana gave their unconditioned contribution to the study and did not have any role in study design, in collection, analysis, and interpretation of data, in the writing of the report, and in the decision to submit the paper for publication.

## Results

**Fig. 1:**
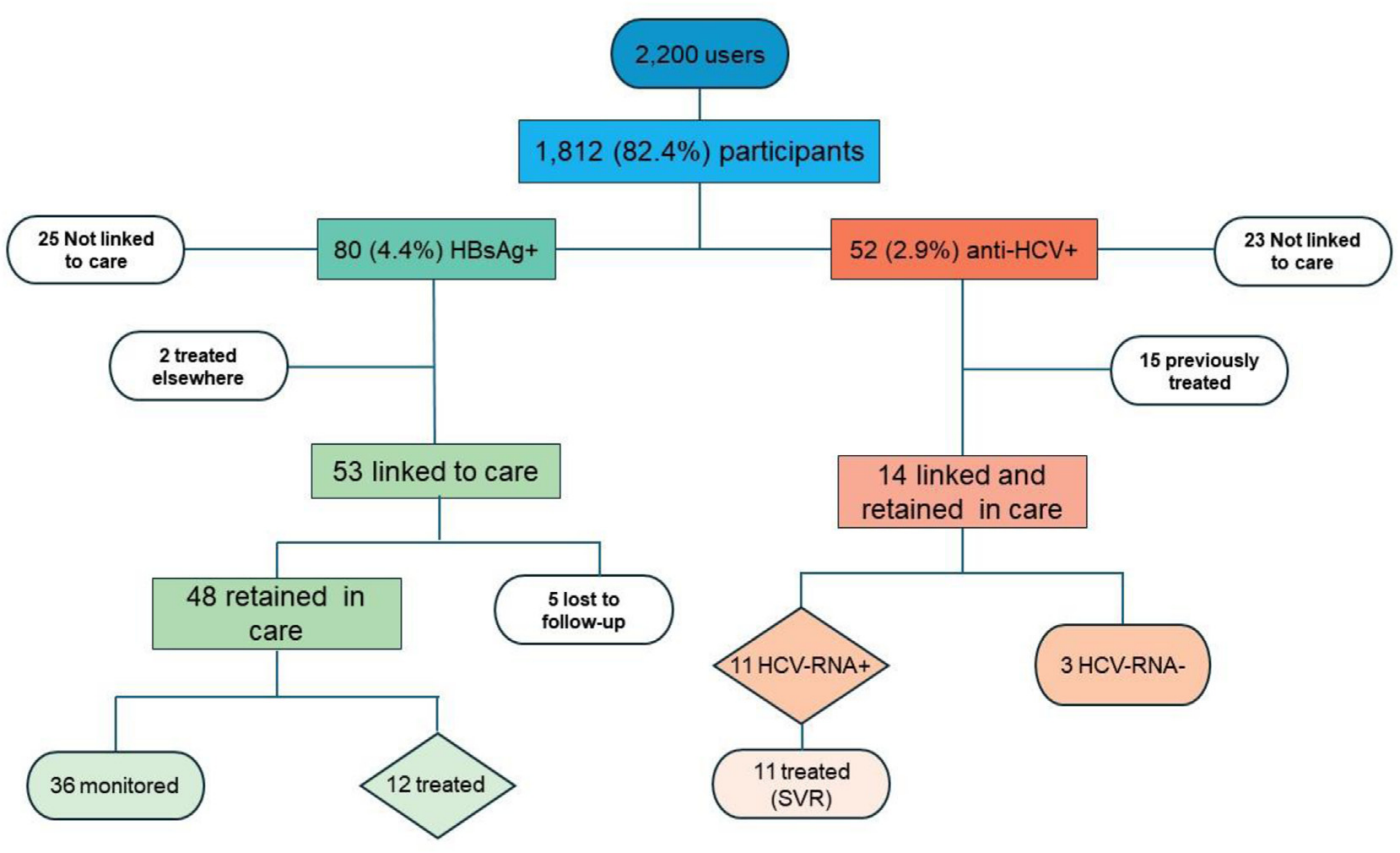
Flow chart summarizing the study outcomes.

From January 2019 to May 2024, a total of 75 testing sessions were performed. The testing activity was stopped during the COVID-19 pandemic from March 2020 to January 2022. Considering the average number of daily users for each charity hosting the sessions, 2203 individuals were approached and invited to participate. Among them, 1812 (82.4%) agreed to HBV and HCV testing as shown in the flow chart reported in Fig. 1.

**Fig. 2:**
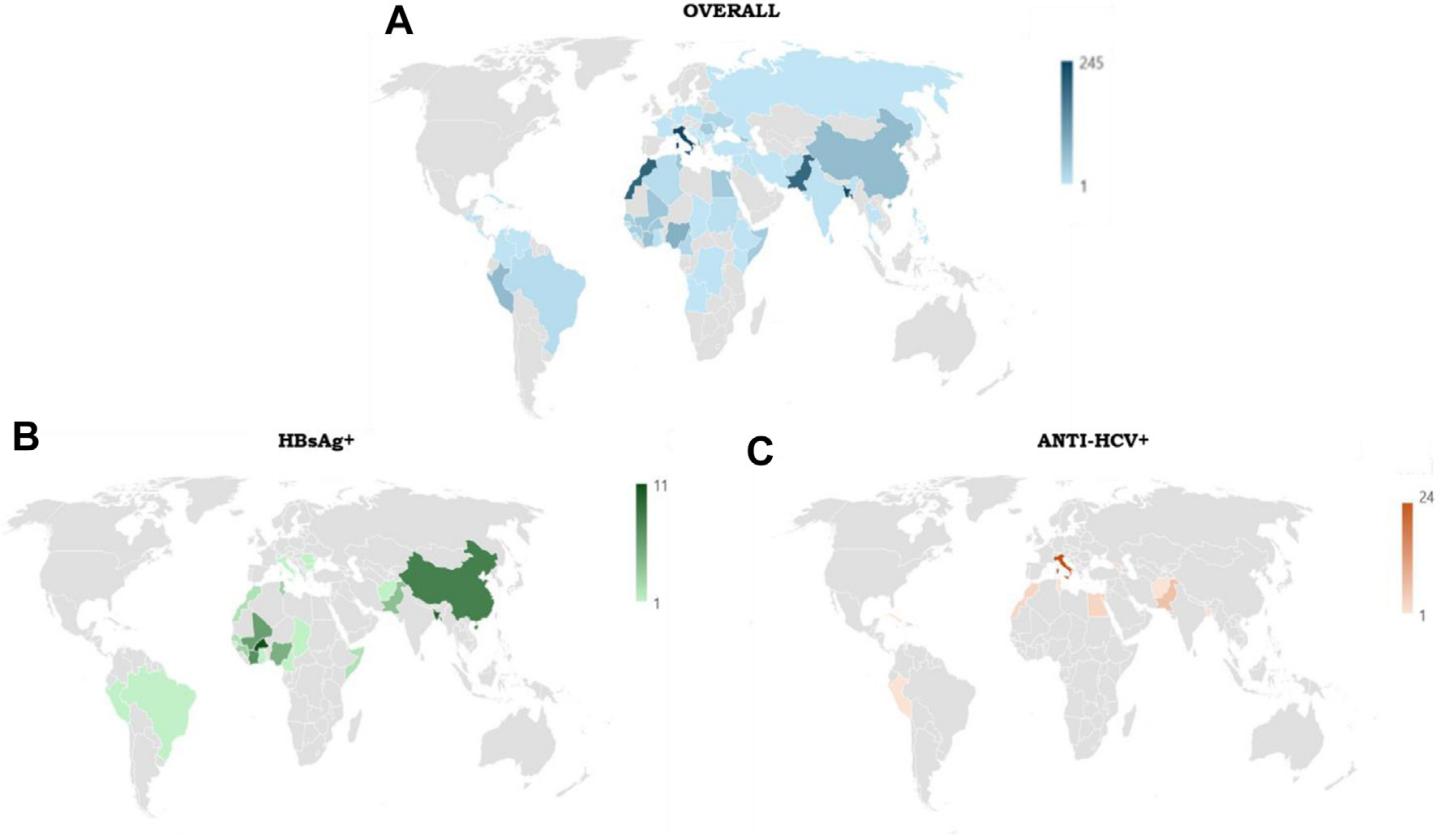
Geographical origin of participants.

The main characteristics of participants, overall and by HBsAg and anti-HCV status, are reported in Table 1. The participants were mostly males (75%), mean age was 38 **±** 14.9 years and Italy was the most represented country of origin (13.5%), followed by Bangladesh (13.1%) and Morocco (11%). The heatmap in panel A of Fig. 2 visually represents the geographical origin of the total population, while the geographical origin of the HBsAg+ and anti-HCV + participants is shown in Fig. 2, panels B and C respectively.

**Table 1:** Characteristics of the study population, overall, and by HBsAg and anti-HCV status.

Characteristic	Total (1812)	HBsAg− (1732)	HBsAg+ (80)	*p*	Anti-HCV− (1760)	Anti-HCV+ (52)	*p*
Age, years, mean ± SD	38 **±** 14.9	38.5 **±** 14.93	33.6 ± 12.4	*p* < *0.001*	38.6 **±** 14.9	49.2 ± 12.6	*p* < *0.0001*
Sex							
Males	1365	1292 (74.6%)	73 (91%)	*p* < *0.001*	1322 (75%)	43 (82.7%)	*p* = *0.4003*
Females	447	440 (25.4%)	7 (9%)		438 (25%)	9 (17.3%)	
Country of origin							
Italy	245	244 (14%)	1 (1.3%)	*p* < *0.001*	218 (12.4%)	27 (51.9%)	*p* < *0.0001*
Others	1567	1488 (86%)	79 (98.7%)		1542 (87.6%)	25 (48.1%)	
Type of charity							*p* < *0.001*
Shelters for immigrants	708	668 (38.5%)	40 (50%)	*p* < *0.05*	701 (39.8%)	7 (13.5%)	
Meal centers	592	564 (32.6%)	28 (35%)		560 (31.8%)	32 (61.5%)	
Food banks	141	140 (8.1%)	1 (1.3%)		135 (7.7%)	6 (11.5%)	
Schools for foreigners	184	177 (10.2%)	7 (8.8%)		182 (10.3%)	2 (3.8%)	
Religious gathering center	93	92 (5.3%)	1 (1.3%)		91 (5.2%)	2 (3.8%)	
Other	94	91 (5.3%)	3 (3.8%)		91 (5.2%)	3 (5.8%)	
Comorbidity							
Hypertension	95	91 (5.3%)	4 (5%)	*p* = *0.3626*	92 (5.2%)	3 (5.8%)	*p* = *0.7417*
Diabetes	79	76 (4.4%)	3 (3.8%)	*p* > *0.999*	76 (4.3%)	3 (5.8%)	*p* = *0.4705*
Dyslipidemia	9	8 (0.5%)	1 (1.3%)	*p* = *0.3346*	9 (0.5%)	0	*p* > *0.999*
Other	316	307 (17.7%)	9 (11.3%)	*p* = *0.1737*	290 (16.5%)	26 (50%)	*p* < *0.0001*
Risk factors							
IV drugs	174	169 (9.8%)	5 (6.3%)	*p* = *0.4324*	143 (8.1%)	31 (59.6%)	*p* < *0.0001*
Surgeries	220	214 (12.4%)	6 (7.5%)	*p* = *0.2866*	213 (12.1%)	7 (13.5%)	*p* = *0.4949*
Blood transfusions	45	42 (2.4%)	3 (3.8%)	*p* = *0.4328*	43 (2.4%)	2 (3.8%)	*p* = *0.3186*
Unprotected sex^a^	510/1361	495/1308 (37.8%)	15/53 (28.3%)	*p* = *0.1927*	493/1323 (38.3%)	17/38 (44.7%)	*p* = *0.3961*
Education^a^							
None	147/1256	136/1210 (11.2%)	11/46 (23.9%)	*p* = *0.087*	146/1211 (11.9%)	1/35 (2.9%)	*p* < *0.01*
Less than high school	534/1256	516/1210 (42.6%)	18/46 (39.1%)		510/1211 (41.8%)	24/35 (68.6%)	
High school	464/1256	449/1210 (37.1%)	15/46 (32.6%)		454/1211 (37.2%)	10/35 (28.6%)	
More than high school	111/1256	109/1210 (9%)	2/46 (4.3%)		111/1211 (9.1%)	0	

HBsAg = hepatitis B virus surface antigen; HCV = hepatitis C virus.

a Data are available for a subgroup of participants as detailed in the table.

Eighty/1812 (4.4%) participants were found to be positive (+) for HBsAg and 52/1812 (2.9%) for the anti-HCV. Two individuals were both HBsAg+ and anti-HCV+.

### HBsAg positivity related outcomes

**Fig. 3:**
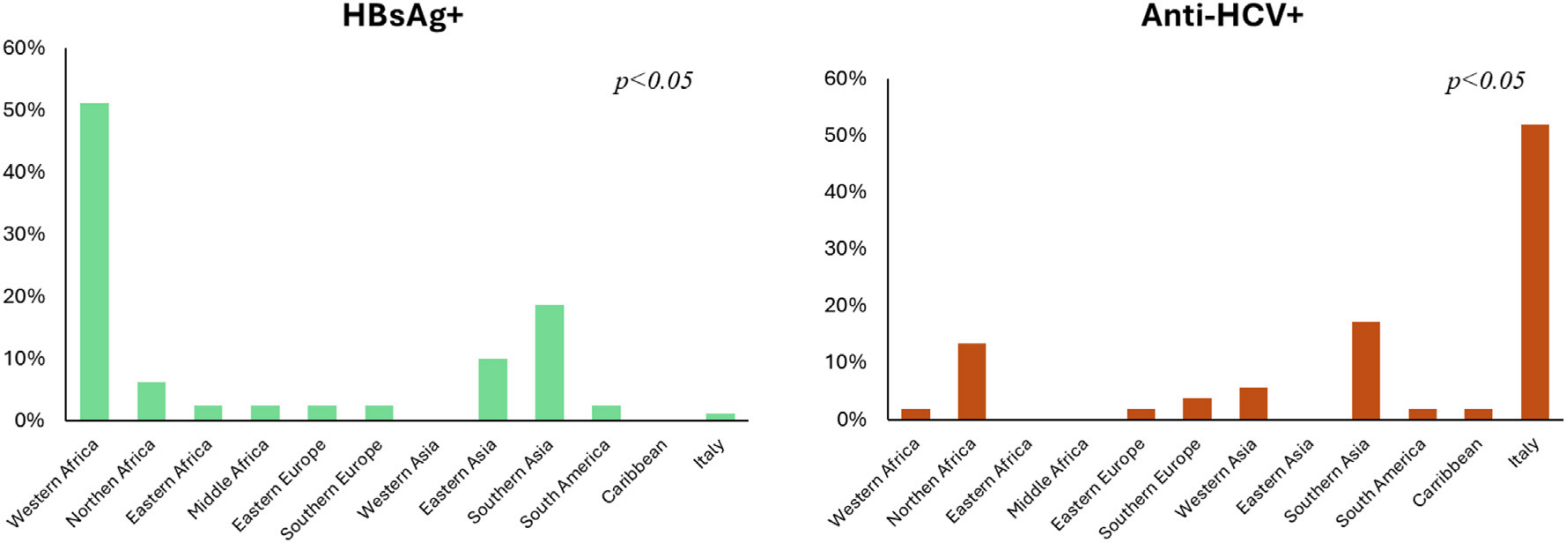
Geographical origin of the HBsAg positive and anti-HCV-positive individuals, based on World regions classified according to the United Nation Geoscheme.

A higher proportion of those who were HBsAg+ were men (91% vs 74.6%; *p* < *0.001*), and non-Italian natives compared to those who were HBsAg- (98.7% vs 86%; *p* < *0.001*). Moreover, HBsAg + individuals had a significantly younger mean age (*p* < *0.001*) (Table 1). The χ^2^ test showed an association with the geographical origins of the participants,^17^ as reported in Fig. 3 (*p* < *0.05*). The analysis of residual showed a significant difference regarding HBsAg positivity (*p* < *0.001*) between participants coming from Eastern Asia (12%) and those coming from Western Asia (no HBsAg + individuals).

Details about the type of charity that hosted the testing sessions, the presence of comorbidity, risk factors and education levels were also reported in Table 1. Overall, 50% of the HBsAg + participants were recent immigrants living in shelters. A significant association between location and HBV positivity (*p* < *0.05*) emerged from a χ^2^ test performed on the hosting charities and test positivity. The analysis of residuals showed higher HBV positivity in “shelters for immigrants” (5.6%, 40/708) and “meal centers” (4.7%, 28/592) compared to “food banks” (0.7%, 1/141) (*p* < *0.01* for both comparisons).

The male sex was found to be the only predictive variable of HBsAg positivity (OR = 3.55, 95% CI: 1.62–7.77, *p* < *0.01*), at regression analysis.

HBsAg + individuals were referred to the closest outpatient clinics. Linkage to care uptake for the HBsAg + participants was 66.3% (53/80), considering that 2 individuals were aware of the infection and already on treatment (2/80, 2.5%) (Fig. 1). The retention in care outcome was reached for 48/53 (90.6%) patients, as 5/53 (9.4%) were lost after the initial clinical assessment visit. The main liver and viral parameters of the retained in care group are reported in Table 2. Fibroscan evaluation was available for 38/48 (79%) and showed overall mild liver damage (Table 2). Twelve/48 (25%) participants were eligible for treatment and started on Entecavir (Table 2), while regular monitoring appointments were scheduled for the remaining patients. All linked-to-care HBsAg + individuals were tested for Human Immunodeficiency Virus (HIV) and Hepatitis D Virus (HDV) infection markers, and no coinfections were found.

**Table 2:** Characteristics of the linked to care HBsAg and anti-HCV positive patients.

Complementary test and administered treatment	HBsAg+ (n = 48)	Anti-HCV+ (n = 14)
Analytical parameter, median (IQR)		
AST, U/L	25 (11–35)	32 (21–34)
ALT, U/L	25 (18–36)	41 (34–59)
GGT, U/L	22 (19–32)	37 (20–92)
Alkaline phosphatase, U/L	57 (55–76)	67 (57–69)
Platelets, 10^3^ U/μL	218 (186–246)	230 (151–260)
Albumin g/dL, mean ± SD	4.4 ± 0.4	4.3 ± 0.3
Bilirubin (mg/dL), mean ± SD	0.7 ± 0.3	0.5 ± 0.3
INR, mean ± SD	1.05 ± 0.1	0.93 ± 0.1
HBV DNA, IU/mL, median (IQR)	2105 (589–9638)	–
HCV-RNA, IU/mL, median (IQR)	–	2,380,000 (931,750–2,655,000)
HCV genotype, n (%)	–	7 (50%)
1a	–	3 (43%)
1b	–	1 (14%)
3	–	3 (43%)
HBV/HCV positivity, n (%)	2 (4%)	2 (14%)
Fibrosis grade (FibroScan®), n (%)	38 (48%)	10 (19%)
F_0_–F_1_	33 (87%)	7 (70%)
F_2_	4 (10%)	1 (10%)
F_3_	1 (3%)	0
F_4_	0	2 (20%)
Anti-HBV treatment, n (%)	12 (25%)	–
Entecavir	12 (100%)	–
Anti-HCV treatment, n (%)	–	11 (79%)
Epclusa	–	6 (55%)
Maviret	–	5 (45%)
SVR, n (%)	–	11 (100%)

HBV = hepatitis B virus; HCV = hepatitis C virus; IQR = interquartile range; AST = aspartate aminotransferase (normal range = 5–45 U/L); ALT = alanine aminotransferase (normal range = 5–45 U/L); GGT = gamma-glutamyl transferase (normal range = 7–55 U/L); AP=Alkaline phosphatase (normal range = 45–115 U/L); Platelets (normal range = 140–450 × 10^3^); Albumin (normal range = 3.6–4.9 g/dL); Bilirubin (normal range = 0.1–1.1 mg/dL); SD = standard deviation; INR = international normalized ratio; SVR = sustained virological response. The distribution of continuous variables was assessed using the Shapiro–Wilk normality test. Continuous variables were reported as mean (SD) if normally distributed, while for not normally distributed variables, data were presented as median (IQR) otherwise.

### Anti-HCV positivity related outcomes

A higher proportion of Italian natives was present among the anti-HCV + participants compared to the anti-HCV- (51.9% vs 12.4%; *p* < *0.001*) (Table 1). Results of χ^2^ test on the geographical origins of the participants^17^ as reported in Fig. 3, showed a significant association with anti-HCV+ (*p* < *0.05*). The analysis of residuals demonstrated a significantly higher HCV positivity rate (11%, 27/245) among participants born in Italy compared to those from Southern Asia (0%, 0/6) and North Africa (2.2%, 7/313) (*p* < *0.05* in both comparisons).

Anti-HCV + individuals had a mean age of 49.2 ± 12.6 years, and 37/52 (71.2%) were younger than baby-boomers (born between 1946 and 1964), a high-risk birth cohort for HCV exposure.

Details about the type of charity that hosted the testing sessions, the presence of comorbidity, risk factors and education levels were also reported in Table 1. The anti-HCV + participants were most likely meal center users. The χ^2^ test indicated a significant association between the location where individuals were contacted and their HCV positivity status (*p* < *0.05*). The analysis of residuals revealed that the proportion of HCV-positive individuals was significantly higher in “meal centers” (5.4%, 32/592) and “food banks” (4.4%, 6/141) compared to “shelters for immigrants” (1%, 7/708) (*p* < *0.05* for both the comparisons).

A previous or current intravenous (IV) drug use significantly correlated with the anti-HCV positivity (*p* < *0.001*) (Table 1). A multivariate logistic regression analysis identified the use of IV drugs as an independent factor for being anti-HCV+ (OR = 16.52, 95% CI: 9.25–29.49, *p* < *0.0001*).

Among the anti-HCV + participants, most individuals had an education level lower than high school (68.6%; *p* < *0.01*) (Table 1). Fifteen/52 (28.8%) were previously treated.

The 37/52 (71.2%) anti-HCV + individuals requiring linkage to care were referred to the closest outpatient clinics but 23/37 (62.2%) refused or did not attend the first clinic appointment. Fourteen/37 (37.8%) patients were linked and further retained in care (Fig. 1). Of the 14 anti-HCV individuals, 3/14 (21.4%) had negative viremia and 11/14 (88.6%) patients had an active infection; all the viremic individuals underwent an antiviral treatment and reached an SVR (Table 2). No HIV coinfections were found in the linked to care anti-HCV + patients.

The major liver and viral data of the 14 anti-HCV + participants are detailed in Table 2. Liver damage evaluated by Fibroscan was available for 10/14 (71.4%) patients and 2/10 (20%) were cirrhotic. Three patients were admitted to a rehabilitation program to treat alcohol addiction (data not shown).

## Discussion

Compared to the general population, marginalized individuals are at higher risk of infectious diseases and may encounter unique barriers in the receipt of health care. Usual screening strategies adopted to reach the WHO goal to eliminate viral hepatitis as a public health threat by 2030, generally do not include disadvantaged people due to problems in the approach, linkage to care, and low compliance. Initiatives focused on underserved populations employing a micro-elimination approach are reported mostly for HCV.^6^^,^^8^ As stated by Lazarus and coworkers, micro-elimination approaches should adhere to specific criteria, although these may need adjustment based on epidemiological contexts and geographic locations.^6^ Despite our study does not meet all the criteria to be defined as a micro-elimination intervention according to Lazarus and colleagues,^6^ the sample size, on-site strategy, and results in terms of participation, prevalence found, and linkage to care could serve as an important model for stakeholders, paving the way for future micro-elimination projects targeting similar populations.

The health inequity of groups of the population, such as migrants and homeless people is a theme of growing interest for the public health community. Screening interventions, such as HBV and HCV testing campaigns, are essential in improving the living conditions of marginalized populations and could help in reducing the high incidence of liver cancer recently reported among people living in economically disadvantaged areas of Europe.^18^

One of the key points to ensure the success of the testing campaign is the method of recruitment as barriers can increase hesitancy regarding testing. Studies analyzing different aspects of this issue can be very useful in repeating successful strategies and improving possible biases. Nevertheless, we have to take into account that every geographical area has a different social context, and screening campaign strategies as well as health needs and barriers in linkage to care and management of the clinical path. This may require an adjustment to the local social environment.

Our study had a high success in terms participation with an 82.4% rate of acceptance to get tested. We chose the onsite strategy: a team of healthcare personnel performed the testing directly at the charity and communicated the result within a short amount of time (usually 10–15 min). Previous studies reported different acceptance rates for screening, depending on the category of the screened population and the strategies employed. A very satisfactory response rate (97.6%) was reported by Sahajian and colleague in Lyon, France.^19^ However, the study sample mostly included participants who consulted health centers of their own accord.^19^ Another successful strategy employed a voucher system of reimbursement for agreeing to undergo testing or completing a survey^20^, ^21^, ^22^ that is especially effective since a major problem for marginalized people is often the lack of money.^23^^,^^24^ Gelberg and colleagues reported an 83% response rate to an HCV exposure testing campaign among homeless adults in Los Angeles, USA where the participants received cash incentives for their involvement.^25^

Overall, despite the fact that we did not offer any reimbursements as the Italian law does not allow it, the participation response to our screening campaign was more than satisfactory. This is likely due to the large number of meals distributed daily by the centers running free meal programs, resulting in a high number of potential participants who can be screened in a short amount of time. Also, recent immigrants who are still not employed, spend most of their time in the shelters where they live and are easily approachable.

In addition, we obtained higher participation percentages in the smaller settings where the volunteers and the director had an established relationship with the majority of users. Previous analyses reported that, in some cases, the relationship with health and social care professionals was a barrier because they treated people experiencing homelessness without empathy.^26^^,^^27^ On the other hand, the improvement of shelter service and staff collaborations in the dissemination of information about infectious disease could assist higher numbers of people to link to health care.^28^ One of the reasons marginalized people avoid tests and treatment is mistrust: in some cases, they perceive that they are not a priority for a shelter’s staff, healthcare providers and social workers.^29^

The HBsAg 4.4% prevalence we reported in the setting of vulnerable individuals is higher than those recorded at national levels on the general population. In fact, Sagnelli et al. stated that only 0.8% of individuals born in Italy were HBsAg+ in 2022^30^ and the Global Health Observatory of the WHO reports a 2022 prevalence of 0.51% in Italy.^31^

Only 1/80 (1.3%) of the HBsAg + participants was born in Italy. This is in line with the progressive reduction of HBV endemicity in our country over the last 50 years due to the universal HBV vaccination since 1991.^30^^,^^32^ Therefore, the 4.4% prevalence of HBsAg we found is due mainly to immigrants who are not vaccinated against HBV as already reported and discussed by Sagnelli et al..^30^ In this regard, the WHO Regional Office for Europe urged the host countries to set up HBV vaccination policies for all unvaccinated migrants. In Italy, the vaccination is accessible and free for anyone who requests it. For this reason, according to the Italian guidelines,^33^ we referred all the HBsAg- individuals to the local disease control and prevention centers to assess if they were susceptible to vaccination and receive the shots if needed.

A very alarming finding is the young age of the HBsAg + participants. This is concerning for the patient liver health as, in this setting, other risk factors for liver damage are frequently concomitant (i.e., alcohol abuse, especially for homeless people), making the hepatic disease rapidly evolving. In addition, it is conceivable that young adults potentially have more opportunities to spread the infection, due to their lifestyle. Since many countries have free vaccination programs, the young age of HBV + confirms, as already discussed, the low compliance to the recommended HBV-immunization, in line with previous results regarding similar settings^34^, ^35^, ^36^, ^37^ and this further stresses the need for dedicated immunization plans.

According to previous reports,^38^^,^^39^ our results showed that HBsAg positivity was more frequent in males.

We could not determine any risk factor associated with HBV infection; thus, the geographical origin from endemic areas with low vaccination compliance emerged as the main cause of HBsAg positivity.

The anti-HCV positivity showed a significant association with Italian nationality. Interestingly, the Italian native sub-population showed a higher prevalence than the one we found in sub-populations of participants coming from high endemicity areas such as North Africa.^40^ Since the vulnerable people are usually unaware of the treatment options, they are not often referred to official healthcare services. The prevalence we observed is even slightly higher than the one of the pre-direct-acting-antivirals era in Italy (2.9% vs 2.3% reported by Sagnelli et al.).^41^ Among the Italian participants, the prevalence of anti-HCV was 11%, nearly five times higher than the percentage reported by Sagnelli and colleagues for the general Italian population,^41^ making the marginalized Italian citizens an ideal target for micro-elimination interventions.

The majority of the anti-HCV + participants were younger than baby boomers, which is the birth cohort with the highest risk for HCV.^42^ This finding supports the National Plan initiated in 2021, which consists of a free-of-charge HCV screening focused on key populations, including current IV drug users attending public drug addiction services, people in prison, and individuals born between 1969 and 1989.^43^^,^^44^ Our results confirm that screening the 1969–1989 birth cohort could effectively discover undiagnosed HCV cases and suggest expanding the screening to include new key populations, such as marginalized people and migrants living in shelters.

The risk factor analysis confirmed that IV drug use was significantly associated with HCV exposure, consistently with previous data.^20^^,^^22^^,^^25^^,^^45^, ^46^, ^47^, ^48^ The majority of anti-HCV + individuals were approached in the meal centers. This is due to the fact that users of free meal centers often experience social exclusion and have risky behaviors in addition to poverty. In other words, in this sub-setting, the higher risk of HCV exposure is due to extremely marginalizing living conditions such as homelessness. This is also corroborated by the significant association of HCV positivity to the use of IV drugs, a habit correlating with advanced marginality.^49^

The linkage to care uptake and retention in care for the HBsAg + participants were successful, even considering the two individuals were already aware of the infection and on treatment. Most of the HBsAg + patients were recruited from shelters for migrants, where they often developed a trusting relationship with navigators. As previously discussed, collaboration with social workers operating in charities is likely the major key to the success of the screening campaigns and subsequent linkage to care. The results of our study, in terms of linkage and retention in care outcomes, reflect this point.

The outcome of linkage to care for anti-HCV + patients needs improvement. Only 14 out of 23 (61%) anti-HCV-positive participants were linked to care, indicating that some individuals with active HCV infection may have been prevented from accessing antiviral therapy. The composition of the anti-HCV + subset explains the reluctance that prevented healthcare access: these individuals, mostly Italians, experienced social exclusion. While recent immigrants perceive their health as an important issue, this is not a priority for extremely marginalized people, for whom social exclusion is a multidimensional phenomenon, not limited to material deprivation.

Therefore, since the definition of marginality is broad and reflects that marginalization is an umbrella term, we can state that HBsAg and anti-HCV positivity are associated with two different subsets among the participants in the testing sessions.

The main hepatic parameters of the retained in care groups showed overall mild liver damage in the HBsAg + participants.

Liver damage evaluation in the anti-HCV individuals showed 2 cirrhotic patients (one with HCC), that were both successfully treated with antiviral therapy. Again, the presence of advanced liver damage among the anti-HCV + patients could be linked to the particular features of this sub-group characterized by habits linked not only to the infection risk but also to a faster evolution of liver damage such as alcohol consumption.

The higher HBV/HCV prevalences we found in marginalized settings compared to the Italian general population, stress the need to implement the screening of vulnerable groups to target residual/hidden infections. Thus, reducing disparities in access to cure and advancing towards the WHO plan to end viral hepatitis as a public health threat by 2030. Results highlighted a difference between HBV and HCV positive sub-settings: while HBV is highly prevalent in economic migrants and refugees temporarily living in shelters and that usually do not experience social exclusion in their countries; the profile of the HCV + individual is that of a person experiencing extreme marginalization, who engages in risky behaviors, and whose priorities do not include their health.

This study has some limitations. Concerning risk factor assessment, 25% of participants declined to respond to the question about “unprotected sex”. Furthermore, the survey regarding education level was introduced later during the study. Thus, data is available for 69% of the participants. Hence missing data was not included in the statistical analysis.

Reasons for non-testing and data on individuals who declined participation were not recorded, which not only prevented analysis of refusal factors but also limited efforts to enhance adherence and compare participants with non-participants.

We detected HCV infection with an RDT which assesses anti-HCV positivity and therefore requires a second test to diagnose an active infection. Conversely, the reflex test assessing viremia has the potential to increase the uptake of HCV testing and linkage to HCV care.

The data about the HCV active infection rate was partial since 9/23 anti-HCV + individuals were not linked to care. This accounts for using tests that reveal the presence of the anti-HCV instead of the reflex care test that detects viral RNA.

Overall, this study found that an on-site HBV and HCV screening strategy can be successful among marginalized communities in Italy, particularly in facilities providing various forms of assistance and support to migrants, homeless individuals, and other vulnerable groups.

Thus, the on-site testing could be a helpful model for European policymakers when reassessing existing health policies and systems regarding viral hepatitis elimination plans. It is essential to develop strategies that enhance healthcare accessibility while fostering trust and engagement with marginalized communities. As demonstrated by the high rate of success we obtained when navigators actively took part in the screening sessions and to the further clinical path, this is only possible through integrated care models. In other words, in this context, successful strategies not only meet medical needs but also address social determinants of health, including housing, employment, and mental health services. Thus, this offers a more comprehensive approach to healthcare by tackling the underlying causes of disparities.

## Contributors

Study design: LG, MM and ALZ. Data collection: LG, MM, TC, AC, IDG, GC, SL, SM, FM, CS, LP, LC, MMA M, LM, EC, AX, IB, SL, LL, SIB, AN, SG, DA, PB. Data analysis: LG, MM, ALZ, MLM. Drafting and revising: LG, MM, GC, ALZ, MLM, SG. All authors have read and approved the final version of the manuscript.

## Data sharing statement

MM and LG have full access to all of the data in the study and take responsibility for the integrity of the data and the accuracy of the data analysis. All data used in the study are available upon request to the corresponding author.

## Editor note

The Lancet Group takes a neutral position with respect to territorial claims in published maps and institutional affiliations.

## Declaration of interests

Stefano Gitto: Gilead, speaker honoraria (2, year 2024), consultant fee (1, year 2024). All other authors declare no competing interests.
